# The HALO Model: A Learning Health System Framework for Artificial Intelligence

**DOI:** 10.1002/lrh2.70100

**Published:** 2026-06-08

**Authors:** Adrian H. Zai, Mohammad Adibuzzaman, David D. McManus, Allan Walkey

**Affiliations:** ^1^ UMass Chan Medical School Worcester Massachusetts USA; ^2^ Oregon Health Science University Portland Oregon USA; ^3^ UMass Memorial Hospital Worcester Massachusetts USA

**Keywords:** AI governance, artificial intelligence, learning health system

## Abstract

**Introduction:**

Artificial intelligence is increasingly embedded in healthcare delivery, yet existing Learning Health System (LHS) models do not fully account for the lifecycle management and continuous assurance requirements of AI systems. This gap limits health systems' ability to safely and sustainably integrate AI as a learning component of care.

**Methods:**

We conducted a conceptual system modeling investigation grounded in LHS theory and contemporary AI governance frameworks. Through structured theoretical integration, we aligned the classical LHS learning cycle with an action‐oriented AI lifecycle and five continuous assurance dimensions, developing a unified framework to support operational implementation within health systems.

**Results:**

The resulting Health AI Learning and Oversight (HALO) model specifies how AI functions as a dynamic knowledge artifact within an LHS. Application of the model illustrates how integrating lifecycle stages and continuous assurance instantiates iterative learning loops, enables adaptive governance, and supports operational lifecycle management, including ongoing monitoring of performance, safety, equity, transparency, and security across clinical environments.

**Conclusions:**

By extending LHS theory to incorporate AI lifecycle and assurance requirements explicitly, the HALO framework operationalizes continuous learning and oversight for AI‐enabled health systems. This model provides a foundation for designing, governing, and sustaining responsible and adaptive AI deployment as healthcare environments evolve.

## 1 Introduction

Artificial intelligence has moved rapidly from the periphery of innovation to the center of healthcare delivery. In this report, we focus primarily on AI systems used for prediction, risk stratification, and workflow support, which represent the dominant forms of clinical AI in use today [1, 2, 3, 4, 5, 6, 7, 8]. While the framework can extend to other modalities such as vision models or agentic systems, these are not the central focus here. Yet the frameworks guiding development and oversight remain fragmented, reflecting a broader misalignment between system‐level learning models and AI governance approaches. AI systems are often built as one‐off projects, evaluated once, and deployed into environments where populations, workflows, and institutional practices continually evolve [9, 10, 11, 12, 13, 14]. These conditions strain governance structures that were never designed to manage systems that continue learning after deployment [9, 14, 15].

The Learning Health System (LHS) [16, 17] offers an appealing foundation for thinking about AI in a broader systemic context. For more than a decade, LHS theory has provided a conceptual foundation for continuous learning in health systems, emphasizing iterative cycles in which data inform knowledge creation and knowledge informs practice changes that generate new data [17, 18, 19]. However, the classical LHS framework predates modern AI systems and does not fully articulate how AI models—which require ongoing monitoring, recalibration, fairness assessment, and risk management—can function as active components of the learning loop [12, 20, 21, 22]. The challenge is not simply to place AI inside the LHS, but to evolve both the LHS and AI so that each reinforces the other's learning processes in a rapidly changing environment [12, 21].

Many health systems still struggle to implement even simple, interpretable tools such as traditional risk scores at scale. The emergence of more complex, less transparent AI models, therefore, raises a natural question: if basic tools remain difficult to sustain, how will we safely implement models that are harder to understand and maintain? Yet the rapid expansion of AI also creates an inflection point, offering a second chance to build the learning and oversight infrastructure that has long been needed but only partially realized.

At the same time, new national frameworks for trustworthy AI, such as those developed by the Coalition for Health AI (CHAI), have introduced important concepts, including lifecycle stages and assurance dimensions [9, 14, 23, 24, 25]. These contributions outline core expectations for responsible AI development but remain only partially integrated into the broader structural and conceptual frameworks that guide how learning occurs in health systems. This highlights an area where integration remains underdeveloped: LHS theory provides an elegant view of system‐wide learning but little about AI development and governance. In contrast, AI governance frameworks describe evaluation steps but rarely account for the systemic and fiscal realities of clinical organizations that aspire to be LHS.

**FIGURE 1:**
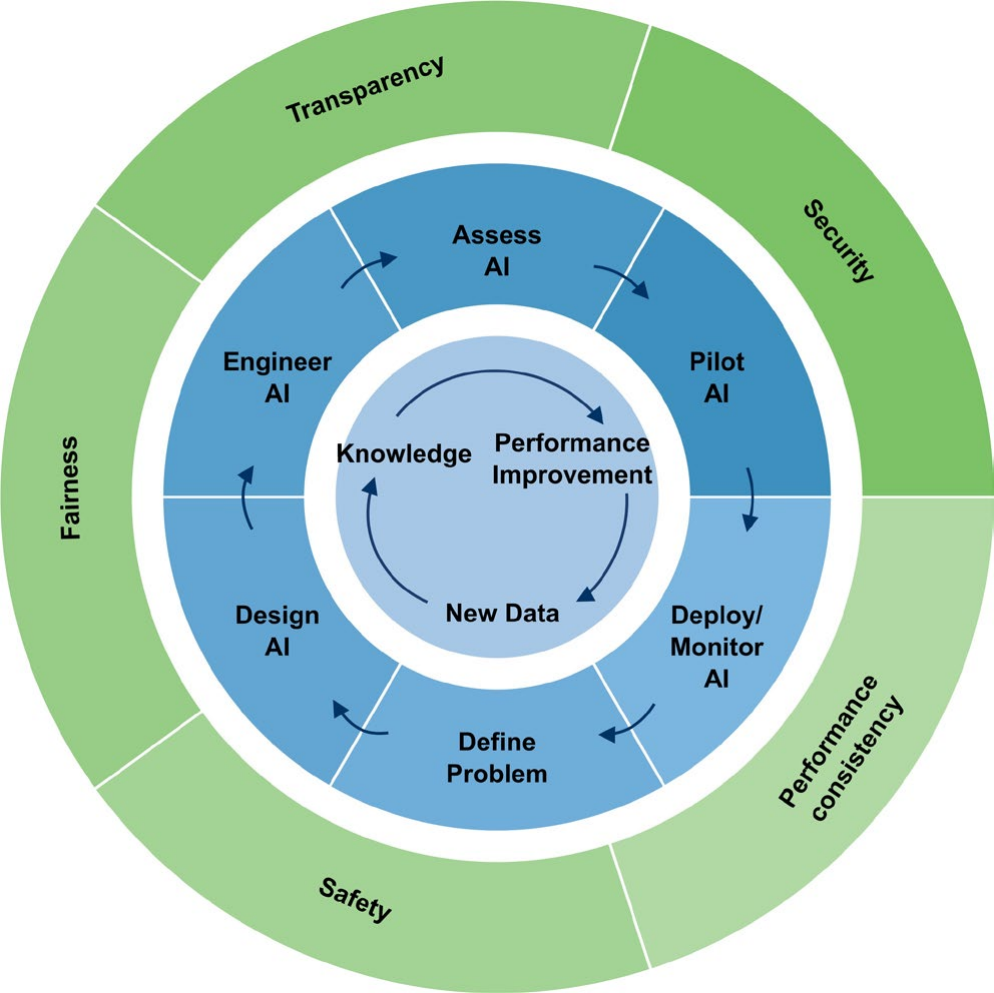
The HALO framework (health AI learning and oversight). The HALO framework integrates three complementary cycles into a unified structure for AI‐enabled Learning Health Systems. The inner loop represents the adapted AI lifecycle, consisting of six action‐oriented stages: Define Problem, Design AI, Engineer AI, Assess AI, Pilot AI, and Deploy/Monitor. These stages describe how interdisciplinary teams develop, evaluate, and iteratively refine AI models in practice. Surrounding this loop is the classical Learning Health System cycle, in which data generate knowledge, knowledge informs performance improvement, and performance improvement produces new data. The outer assurance layer reflects continuous oversight across five dimensions that influence the safe and trustworthy use of AI in health systems. Together, these layers illustrate how AI development, model governance, and system‐level learning operate as an integrated process, allowing both the health system and the AI model to evolve in response to real‐world evidence.

We propose the HALO (Health AI Learning and Oversight) model, a system‐level conceptual framework that integrates LHS principles with an adapted AI lifecycle and a continuous assurance layer, and supports the operationalization of AI within LHS. The HALO model builds on the classical LHS while incorporating the lifecycle and oversight requirements unique to AI systems, treating AI as a continuously learning and supervised component of the health system (see Figure 1). The framework updates the classical LHS concept for the AI era, positioning artificial intelligence as a dynamic knowledge artifact that must be continuously monitored, contextually aligned, and embedded within a system that learns alongside it. The goal is not simply to outline how AI should be governed but to describe how AI can function as a core component of a continuously evolving LHS. HALO operationalizes key elements emphasized in CHAI by embedding them directly into the LHS's continuous learning processes.

### 1.1 Foundations in LHS Theory

The concept of a LHS has provided a powerful organizing principle for modern healthcare reform. In its traditional form, the LHS describes a continuous cycle that begins with clinical care generating data. These data are aggregated and analyzed to generate new knowledge, which then informs improvements in practice [17]. When implemented, these improvements produce new data, which feed the next iteration of learning. This cyclic structure allows health systems to evolve based on real‐world evidence, emerging insights, and observed performance. The LHS is a sociotechnical construct involving people, processes, data flows, governance structures, and organizational culture [26, 27, 28, 29, 30, 31, 32, 33, 34]. When functioning well, the LHS creates conditions in which clinical practice evolves through a dynamic interplay between evidence generation and practical implementation.

However, the arrival of AI introduces complexities that the classical LHS was not designed to manage. AI systems produce new forms of knowledge, often in the form of predictive outputs or latent representations, and these outputs may influence care directly. Unlike static clinical evidence, both AI models and traditional prediction tools can drift or lose calibration as patient populations and practice patterns evolve. The difference is that AI makes this challenge more visible, creating an opportunity to address a problem that has long existed but has often gone unnoticed in non‐AI tools [35, 36, 37]. LHS frameworks already emphasize iterative monitoring and reassessment, but AI heightens the need to apply these processes consistently and more frequently. Algorithmic knowledge changes more rapidly than traditional evidence, so the LHS must operationalize its learning cycle with greater rigor to preserve safety, fairness, and effectiveness.

### 1.2 Limitations of Traditional AI Governance

Much of today's AI governance remains anchored to episodic evaluation frameworks [38, 39, 40]. Models are often developed, validated once on retrospective data, and deployed into workflows where real‐world performance may diverge from expectations. These divergences can arise from changes in patient mix, clinical practice, workflows, or documentation. Many health systems lack monitoring infrastructure, leaving models without adequate oversight.

The fragmented nature of governance is another barrier [9, 41, 42]. AI models often originate within analytic teams with limited ties to the clinicians or operational staff who will ultimately rely on them. Conversely, clinicians may lack visibility into how models were trained or validated, inhibiting trust and informed use. Compliance teams frequently lack mechanisms to assess ongoing fairness, security, or safety risks, particularly after models enter production environments. These limitations create vulnerability and highlight the need for an integrated learning architecture.

### 1.3 Extending the LHS for the AI Era

Integrating AI into a health system requires viewing it not as a static product but as a component that must learn and adapt within an evolving environment. Because workflows, populations, and documentation shift over time, model performance naturally changes.

These dynamics reflect the fact that healthcare functions as a complex adaptive system, where autonomous agents interact through nonlinear and often unpredictable feedback loops. Introducing AI adds a new actor to this environment, making downstream effects hard to forecast. In such contexts, the Cynefin framework underscores the need to probe, sense, and respond as patterns emerge [18, 43]. An LHS provides this structure.

Extending the LHS for the AI era, therefore, requires making its learning cycle operational for algorithmic tools. Models must be continuously monitored and refined, and the LHS must incorporate mechanisms for governance and assurance so that algorithmic knowledge remains aligned with clinical needs, ethical expectations, and system priorities as both the AI and the environment evolve.

In this work, we propose the HALO model as a system‐level conceptual framework that integrates LHS principles with an adapted AI lifecycle and a continuous assurance layer. The HALO model explicitly links AI lifecycle execution to system‐level learning processes and embeds continuous governance as a core property of the system rather than an external function. This work contributes a unified conceptual structure that aligns AI development, deployment, and oversight with the iterative learning processes that define LHSs.

## 2 Methods

### 2.1 Model Development and Theoretical Integration

The HALO model was developed through a structured process of theoretical integration and system modeling, grounded in established LHS theory and emerging national guidance on trustworthy artificial intelligence, and informed by review of foundational LHS literature and contemporary AI governance frameworks, including widely cited conceptual models, architectural frameworks, and recent synthesis studies [18, 19, 28, 44, 45, 46, 47, 48] alongside guidance such as the CHAI Blueprint. Foundational LHS constructs describing cyclical learning through data generation, knowledge production, and performance improvement were identified from this body of conceptual and empirical literature. In parallel, contemporary AI lifecycle frameworks emphasizing development, validation, deployment, and postdeployment monitoring were identified through national guidance documents and peer‐reviewed literature on trustworthy AI, with particular attention to guidance articulated by the CHAI. These frameworks were then systematically mapped by the study team to identify correspondences and misalignments between system‐level learning processes and how AI is typically developed and governed. This mapping involved aligning key components of the LHS cycle (data generation, knowledge production, and practice change) with stages of the AI lifecycle, followed by iterative refinement to ensure conceptual consistency and relevance to clinical and operational contexts. Through iterative synthesis, we defined an action‐oriented AI lifecycle that preserves the intent of existing AI governance frameworks while aligning more closely with how interdisciplinary clinical, informatics, and operational teams conceptualize and execute AI development in practice. This process was conducted by the authors (A.H.Z., M.A.) through iterative discussion and consensus and is consistent with established methods for conceptual model development in health systems research, emphasizing theory integration and iterative refinement rather than empirical hypothesis testing.

Finally, continuous assurance dimensions, including performance consistency, safety, fairness, transparency, and security, were integrated as cross‐cutting constructs applied to each stage of the lifecycle, rather than as discrete evaluation checkpoints. The resulting model specifies a unified structure in which AI functions as a dynamic knowledge artifact within an LHS, with explicit feedback loops connecting model development, deployment, monitoring, and system‐level learning over time.

## 3 Results

### 3.1 An Adapted AI Lifecycle for an LHS

The HALO model comprises three integrated components: an adapted AI lifecycle, the LHS cycle, and a continuous assurance layer (Figure 1). Table 1 illustrates how the adapted HALO lifecycle stages align with core LHS functions and contribute to system‐level learning.

**TABLE 1:** Alignment of AI lifecycle stages with learning health system functions in the HALO model.

HALO AI lifecycle stage	Corresponding LHS function	Role within HALO model	Illustrative outputs
Define problem	Data generation and problem identification	Identifies clinical or operational gaps using real‐world data and stakeholder input; defines intended use, population, and workflow context	Problem statements, use case definitions, target populations, success criteria
Design AI	Knowledge planning and specification	Translates the defined problem into a modeling approach, including selection of data sources, features, model types, and governance considerations	Model design specifications, feature sets, data schemas, governance plans
Engineer AI	Knowledge generation	Develops and trains the AI model, iteratively refining algorithms and features to produce actionable knowledge artifacts	Trained models, model parameters, feature importance, training pipelines
Assess AI	Evidence evaluation	Evaluates model performance, robustness, calibration, fairness, and failure modes prior to real‐world use	Performance metrics, validation reports, subgroup analyses, risk assessments
Pilot AI	Practice testing and implementation	Tests the model in real‐world or simulated workflows to assess usability, integration, and sociotechnical interactions	Pilot study results, workflow impact assessments, user feedback, implementation insights
Deploy and monitor AI	Performance improvement and continuous learning	Integrates the model into clinical workflows and continuously monitors performance, drift, safety, and equity, triggering iterative updates	Clinical decision support outputs, monitoring dashboards, drift detection signals, audit logs, model updates

While the CHAI Blueprint outlines a lifecycle for responsible AI that includes assessment, planning, development, validation, deployment, and monitoring, health systems often require terminology that more directly reflects how interdisciplinary teams conceptualize and execute their work. To support this alignment, we propose an adapted, action‐oriented lifecycle consisting of six stages: Define Problem, Design AI, Engineer AI, Assess AI, Pilot AI, and Deploy/Monitor [24]. These stages are consistent with CHAI's intent but offer clearer resonance with clinical, informatics, and operational stakeholders. This framework is intended to guide implementation while remaining adaptable to the local institutional context.

Define Problem clarifies intended use, population, context, and workflow fit. Design AI specifies modeling approaches, data sources, features, and oversight pathways. Engineer AI develops and refines the model. Assess AI evaluates validity, robustness, calibration, fairness, and failure modes. Pilot AI examines workflow integration and sociotechnical interactions. Deploy/Monitor supports real‐world implementation while ensuring continuous surveillance for drift and enabling updating or recalibration.

The Deploy/Monitor stage generates real‐world data that initiates subsequent iterations of problem definition, creating a learning loop structurally analogous to the classical LHS cycle. This alignment ensures that each AI lifecycle iteration functions as a learning cycle nested within, and contributing to, the broader LHS framework. By framing these activities as cyclical rather than linear, the adapted lifecycle supports continuous learning for the AI model and the health system in which it is embedded.

### 3.2 AI Assurance as a Continuous Governance Layer

Continuous assurance forms the outermost layer of the HALO framework. Drawing from CHAI and related national guidance, five assurance dimensions inform every stage of the AI lifecycle: performance consistency, safety, fairness, transparency, and security. These dimensions are not discrete checkpoints but ongoing conditions that shape decisions about model progression, modification, or withdrawal throughout the model's operational life.

Assurance plays a critical role in maintaining trustworthiness. Performance consistency ensures reliable function across populations and over time. Safety addresses risks of harm, including inappropriate recommendations and unintended workflow interactions. Fairness requires equitable performance across demographic groups and mitigation of structural bias. Transparency supports informed use by clarifying how models function and should be interpreted. Security protects model integrity and guards against malicious interference. By embedding these dimensions across the lifecycle, the framework positions trustworthiness as a continuous requirement rather than a predeployment achievement.

### 3.3 Integrating the LHS Cycle, AI Lifecycle, and Assurance Dimensions

The HALO framework synthesizes the LHS cycle, the adapted AI lifecycle, and continuous assurance into a unified system of learning and governance. At the center lies the LHS cycle, in which data generate knowledge and knowledge informs practice. The adapted lifecycle operationalizes how AI models are defined, developed, evaluated, piloted, and monitored within this loop. The assurance layer shapes the conditions under which these processes occur.

This nested architecture reflects the reality that AI operates within, and must remain aligned with, broader system‐level learning processes. AI lifecycle stages are embedded within the LHS cycle: model development corresponds to knowledge generation, deployment aligns with practice integration, and monitoring feeds directly into system‐level learning. Continuous assurance functions as a cross‐cutting layer that informs decisions at each stage, determining whether models advance, are modified, or are withdrawn. Within this structure, AI is positioned not as an autonomous decision‐maker but as a knowledge artifact that evolves through iterative learning, guided by human expertise, regulatory expectations, and institutional priorities.

### 3.4 Application of the HALO Model to SSI Surveillance

Applying the HALO framework to a machine learning‐based surgical site infection surveillance use case illustrates how integrating the AI lifecycle within an LHS fundamentally alters how learning, governance, and adaptation occur over time [49]. Rather than treating model development and deployment as discrete events, the HALO model makes explicit how each stage of the AI lifecycle generates system‐level learning and triggers subsequent cycles of improvement.

During problem definition, quality reports and clinician feedback function as signals within the LHS that identify gaps in detection practices and equity considerations. These inputs establish a learning objective that is revisited iteratively. During design and engineering, interdisciplinary collaboration produces models optimized not only for predictive performance but also for interpretability, workflow integration, and downstream assurance requirements.

Assessment activities instantiate the knowledge phase of the LHS by generating evaluative outputs such as calibration performance, subgroup behavior, and failure modes that inform governance decisions. Rather than serving solely as a deployment gate, assessment results shape pilot scope, workflow modifications, and human oversight strategies. The pilot phase further extends learning by revealing sociotechnical interactions and enabling clinician feedback to inform both model refinement and care process redesign.

Following deployment, continuous monitoring operationalizes the performance improvement phase of the LHS by detecting drift, workflow impacts, and emerging equity concerns. These signals inform decisions about recalibration, retraining, or decommissioning. Monitoring outputs generate new data that directly initiate subsequent iterations of problem definition, closing the learning loop.

Compared with traditional AI implementations, which often lack structured feedback pathways, the HALO model produces a system characterized by continuous recalibration, shared accountability, and adaptive governance. In this way, HALO transforms AI from a static analytic artifact into a dynamic component of system‐level learning.

## 4 Discussion

This work contributes to the LHS literature by extending its conceptual foundation to explicitly incorporate artificial intelligence as an active component of system‐level learning. The HALO model integrates AI lifecycle processes with LHS feedback loops and reframes governance as a continuous, embedded function rather than an episodic evaluation activity. In doing so, it provides a structured and operationally relevant framework for aligning AI development, deployment, and oversight with the iterative learning processes that define LHS. While prior LHS models have focused on cyclical knowledge generation and practice improvement, they have not fully specified how AI systems should be managed as continuously evolving components within these cycles. The HALO framework addresses this gap by integrating AI lifecycle execution and continuous assurance directly into system‐level learning processes, positioning governance as an intrinsic feature of the learning system.

### 4.1 Organizational Readiness and Infrastructure for AI‐Enabled LHSs

Implementing the unified framework requires meaningful organizational investment. Data infrastructure must support timely ingestion, secure storage, and integration across systems. Governance structures must oversee the lifecycle and enable collaboration among clinical, data science, informatics, and operational teams. Workforce competencies must be developed so that clinical and analytic staff understand AI's capabilities and limitations. Sociotechnical alignment is essential; AI must support workflows, and teams must provide feedback for continuous refinement.

These capabilities are fundamental to the framework's success, as without them the AI lifecycle cannot be embedded in the LHS or support continuous assurance. For example, an institution operationalizing HALO could implement a centralized AI governance structure that integrates model registry capabilities, real‐time performance monitoring dashboards, and interdisciplinary review processes. Within such a structure, models would progress through defined lifecycle stages with embedded checkpoints for safety, fairness, and performance, while monitoring outputs continuously inform recalibration or retraining decisions. This type of infrastructure enables the HALO framework to function as an operational system rather than a purely conceptual model. In this configuration, outputs from model monitoring and evaluation feed directly into governance processes and subsequent problem redefinition, ensuring that each stage of the lifecycle contributes to continuous system‐level learning.

By making explicit the connections between AI lifecycle stages, system‐level learning processes, and continuous assurance, the HALO model provides a practical foundation for operationalizing AI within health systems. Rather than treating model evaluation as a one‐time event, the framework supports ongoing monitoring, feedback, and adaptation, enabling health systems to respond to performance drift, changing populations, and evolving clinical contexts. This has implications for how organizations design infrastructure, allocate responsibility, and integrate AI into clinical workflows.

The model also has implications for governance. By embedding assurance as a continuous, system‐level function, HALO shifts oversight from periodic review toward ongoing accountability. This approach aligns governance with real‐world conditions in which AI systems evolve over time and require continuous evaluation across multiple dimensions, including safety, fairness, and performance.

Sustaining a HALO‐style engine also requires ongoing investment. Continuous learning and assurance depend on reliable data pipelines, analytic and informatics expertise, governance time, and clinical engagement. While a full economic analysis is beyond the scope of this report, recognizing the financial and operational costs of running AI as a learning component of an LHS is essential for realistic planning and adoption.

### 4.2 Scaling Across Institutions and Federated LHSs

As health systems increasingly collaborate across regional or national networks, the unified model must scale across institutions with diverse populations and workflows. Multisite implementation raises new challenges, including variability in data quality, heterogeneity in documentation patterns, and the potential for differential performance across populations. Federated LHS networks can support collaborative learning by allowing models to be evaluated or trained across multiple institutions without requiring centralized data aggregation [50].

Federated learning provides a natural architectural extension of the LHS by enabling model training, evaluation, and updating across institutions while keeping data local. This approach maintains institutional control over sensitive information, supports privacy‐preserving collaboration, and allows learning cycles to occur across heterogeneous populations. Federated learning also enables multisite assurance workflows, including subgroup fairness evaluation, drift detection, and cross‐context robustness testing. By allowing knowledge to flow while data remain local, federated learning operationalizes the LHS vision at regional and national scale [50].

### 4.3 Equity and Community Implications

Equity is central to the responsible integration of AI in healthcare. AI models can inadvertently perpetuate or amplify structural inequities if they are trained on biased data or deployed in contexts that differ from their training environments [51, 52, 53, 54, 55]. Embedding AI within an LHS enables continuous monitoring of subgroup performance and supports early identification of inequities.

Community engagement is essential to ensure that model design, deployment, and monitoring reflect the values and needs of patient populations [56, 57, 58]. LHS learning extends to both clinical workflows and community relationships. An equity‐oriented LHS explicitly supports feedback loops that elevate community knowledge and guide improvements in both care quality and model design.

### 4.4 Future Landscape and Emerging Directions

The future of AI in healthcare will extend beyond current prediction models to include generative [59], multimodal [60, 61, 62], and adaptive systems [15, 63]. These technologies will increase the complexity of monitoring, evaluation, and regulatory oversight, underscoring the need for LHS‐aligned structures that support continuous assurance and system‐level learning.

Regulatory agencies are already signaling these transitions [64]. The FDA has emphasized the importance of lifecycle monitoring and postmarket surveillance for adaptive AI systems [9, 65, 66]. National and international bodies are developing frameworks that align AI governance with broader principles of safety, equity, transparency, and accountability [67, 68, 69, 70, 71]. The HALO framework presented here provides a conceptual and operational foundation for harmonizing institutional practices with emerging regulatory expectations.

At the same time, implementing this model presents practical challenges. Many health systems lack the data infrastructure, monitoring capabilities, and interdisciplinary coordination required to support continuous learning and assurance. As a result, HALO should be understood as an aspirational framework that can be implemented incrementally, with organizations adopting components based on their maturity level and available resources.

### 4.5 Limitations

This work presents a conceptual, system‐level model for integrating artificial intelligence within LHSs and does not include empirical validation in a specific clinical setting. The HALO framework assumes the presence of foundational LHS capabilities, including robust data infrastructure, interdisciplinary governance, and a workforce equipped to support continuous learning. These capabilities vary widely across organizations, which may limit immediate applicability or require adaptation in less mature environments.

The framework emphasizes system‐level learning processes, feedback loops, and continuous assurance rather than optimization of individual model performance. It does not prescribe specific technical architectures, implementation pathways, or economic strategies, which are likely to differ based on institutional context, regulatory requirements, and available resources.

As a result, HALO should be understood as a guiding structure rather than a prescriptive solution. Future empirical studies across diverse healthcare settings will be necessary to evaluate how different implementations of the framework influence safety, equity, performance, and sustainability over time.

## 5 Conclusion

AI has the potential to accelerate learning and improve clinical outcomes, but realizing this promise requires embedding AI within a system capable of continuous evaluation and improvement. Many health systems still struggle to implement even simple prediction tools at scale, raising the stakes for more complex and less interpretable models. Without a stronger learning and oversight infrastructure, AI's benefits will be difficult to achieve.

At the same time, the rapid expansion of AI offers an opportunity to build that infrastructure more deliberately. By extending the LHS to include an adapted AI lifecycle and continuous assurance, the HALO framework positions AI as an evolving, supervised knowledge artifact that participates in an ongoing learning process within a complex adaptive system.

This report does not prescribe a single implementation pathway, and operational and financial requirements will vary across institutions. Instead, it offers a conceptual roadmap that unifies LHS principles, AI lifecycle methods, and continuous assurance. We hope that this integrated view supports health systems in deploying AI in ways that are sustainable, equitable, and resilient as clinical and operational environments evolve, and to support the safe, continuous integration of AI into routine care.

## Author Contributions

A.H.Z. conceived the overall framework, led the theoretical integration, developed the narrative structure, and drafted the manuscript. M.A. contributed expertise in computer science, AI lifecycle methodology, and implementation considerations across distributed learning environments, and provided critical revisions to ensure technical rigor. D.D.M. contributed expertise in clinical informatics and implementation and critically reviewed the manuscript. A.W. provided expertise in Learning Health Systems and contributed to the theoretical grounding and manuscript revisions. All authors reviewed and approved the final version of the manuscript.

## Funding

This work was partially supported by the Center for Clinical and Translational Science, University of Massachusetts Grant # (1UM1TR005454‐01A1).

## Conflicts of Interest

The authors declare no conflicts of interest.

## Data Availability

Data sharing not applicable to this article as no datasets were generated or analysed during the current study.
